# Revealing Grain Boundary Sliding from Textures of a Deformed Nanocrystalline Pd–Au Alloy

**DOI:** 10.3390/ma11020190

**Published:** 2018-01-25

**Authors:** Laszlo S. Toth, Werner Skrotzki, Yajun Zhao, Aurimas Pukenas, Christian Braun, Rainer Birringer

**Affiliations:** 1 Laboratory of Excellence on Design of Alloy Metals for Low-Mass Structures (DAMAS), Université de Lorraine, 57073 Metz, France; yajun.zhao@univ-lorraine.fr; 2 Laboratoire d’Etude des Microstructures et de Mécanique des Matériaux (LEM3), Université de Lorraine, 57073 Metz, France; 3 Institut für Festkörper- und Materialphysik, Technische Universität Dresden, D-01062 Dresden, Germany; skrotzki@mail.zih.tu-dresden.de (W.S.); aurimas.pukenas@tu-dresden.de (A.P.); 4 Experimentalphysik, Universität des Saarlandes, D-66041 Saarbrücken, Germany; c.braun@nano.uni-saarland.de (C.B.); ph12rb@zimbra.uni-saarland.de (R.B.)

**Keywords:** Pd–10Au alloy, shear compression, texture, grain boundary sliding

## Abstract

Employing a recent modeling scheme for grain boundary sliding [Zhao et al. *Adv. Eng. Mater.* **2017**, doi:10.1002/adem.201700212], crystallographic textures were simulated for nanocrystalline fcc metals deformed in shear compression. It is shown that, as grain boundary sliding increases, the texture strength decreases while the signature of the texture type remains the same. Grain boundary sliding affects the texture components differently with respect to intensity and angular position. A comparison of a simulation and an experiment on a Pd–10 atom % Au alloy with a 15 nm grain size reveals that, at room temperature, the predominant deformation mode is grain boundary sliding contributing to strain by about 60%.

## 1. Introduction

As grain size decreases, the mechanical properties of polycrystalline materials become increasingly determined by their grain boundaries. This grain-boundary-mediated plasticity dominates at grain sizes below approximately 20 nm [1], leading to “grain size softening”, also called “inverse Hall–Petch behavior” [2]. Moreover, texture formation is significantly reduced [1,3]. These characteristic features indicate a kind of superplastic behavior dominated by grain-boundary-mediated deformation. How this deformation process actually takes place is not clear so far. For nanocrystalline (nc) materials, grain-boundary-mediated deformation is considered a cooperative shearing process in which several grains are involved [4]. Another approach is to assign the grain boundaries to an amorphous structure in which shear shuffling or shear transformation takes place [5]. By applying general terminology, this process is simply called grain boundary sliding (GBS) here. It should also be noted that, besides GBS, grain boundary migration also takes place, leading to grain growth due to stress-induced shear-coupling [1,6].

Recently, a new GBS model was presented and applied successfully on the texture evolution of a Pd–10 atom % Au nc alloy [7]. That model is applied in the present work using experimental results published recently on the same alloy [5] for a different strain path. By comparing experimental and simulated texture results, the amount of GBS was estimated.

## 2. Experimental Details

### 2.1. Material, Deformation, and In-Situ X-ray Microdiffraction

The material system studied was an nc Pd–10 atom % Au alloy prepared by inert-gas condensation followed by powder compaction at a pressure of 2 GPa to disk-shaped samples with a diameter of 8 mm and a thickness in the range of 0.5–1.0 mm [8]. The grain size distribution was lognormal and the texture was almost random [1]. The area-averaged grain size determined by X-ray line profile analysis was about 15 nm [1]. Note that the Pd–10 atom % Au alloy is a solid solution with a relatively high stacking fault energy of 150 mJ·m^−2^ [3].

**Figure 1:**
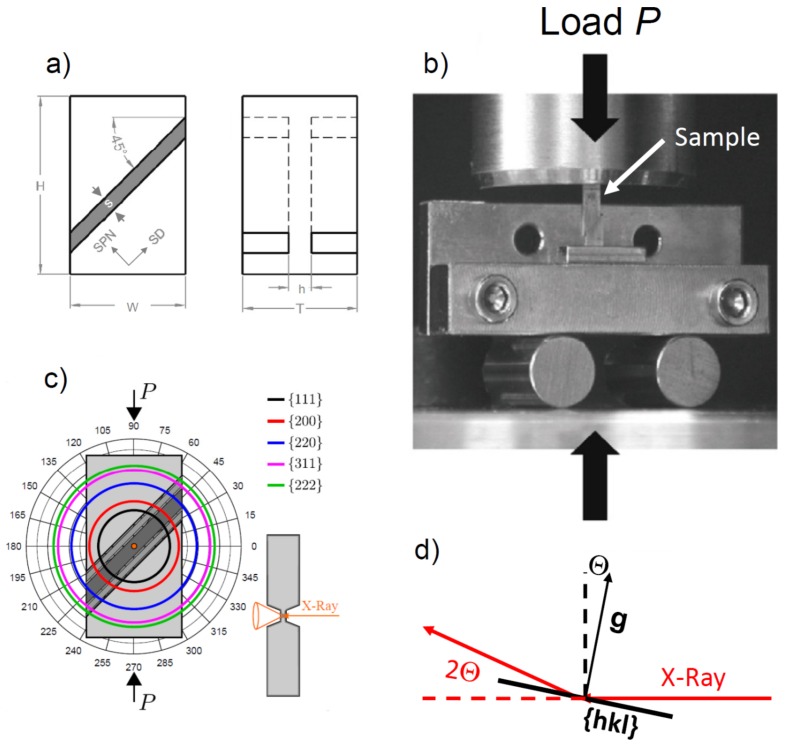
(**a**) Shear compression specimen in front and side view with dimensions marked; (**b**) experimental setup of shear compression; (**c**) sketch of a shear compression specimen (SCS) in front and side view serving as reference frame for obtaining polar plots. In all cases, the slit of the SCS is positioned 45° from the lower left to the upper right, and load is applied along the vertical direction. The smaller side view shows the trapezoid slit geometry. (**d**) Sketch explaining the deviation of the diffraction vector g from the position normal to synchrotron beam corresponding to an outer great circle in the pole figures.

Deformation was carried out on a small-scale specimen, the specific geometry of which is shown in Figure 1. Such a specimen is termed shear compression specimen (SCS) [9] and was cut by spark erosion from the as-prepared disk. Two parallel and oblique slits recessed on opposing faces formed the gauge section. The dimensions of the SCS were 7 mm × 0.95 mm × 0.77 mm (H × W × T). The gauge section was thinned to *h* = 123 µm with a width of about *s* = 120 µm (Figure 1a). Applying a compression stress, the SCS was forced to shear along the gauge section oriented at 45° with respect to the compression axis (Figure 1b). Since plastic deformation is confined to the gauge section [10], the SCS is ideally suited for synchrotron radiation-based transmission experiments. Such experiments were done at the high energy micro-diffraction end-station of beamline ID15A at the European Synchrotron Radiation Facility (ESRF, Grenoble, France). Mechanical testing was performed by a tension/compression device from Kammrath–Weiss (Dortmund, Germany) made up of two load plungers moving symmetrically towards the center, thus keeping the gauge section in a fixed position relative to the incoming beam. The lower plunger was replaced by a roll bearing wagon to substantially reduce the friction coefficient of the horizontal sample movement that goes along with the enforced shear deformation (Figure 1b) [10]. The applied strain rate was about 10^−3^ s^−1^.

The sample deformation was recorded by a CCD camera from Pixelink (Ottawa, ON, Canada) using an inline microscope lens for magnification and several light sources to illuminate the sample. The images were processed by digital image correlation for extracting true sample displacements to establish load–displacement curves. As the gauge section of the SCS deforms under dominant simple shear but also under an appreciable superimposed plane strain compression, there are no simple formulas to compute stress–strain curves from load–displacement data. Instead, finite element analysis was employed using Abaqus to generate load–displacement curves that best approximated the experimental curves. More details on SCS testing are given in [5,11,12].

The testing device with the installed SCS was mounted on top of a six-axis goniometer, which enables the exact placement of the sample with respect to the incoming synchrotron beam. The focused beam with a cross section of 8 µm × 20 µm was directed to the center of the thin gauge section, thus penetrating approximately 30 billion grains. The high energy X-ray radiation had a wavelength of λ = 0.0178 nm. To avoid shadowing of the scattering cones by the edges of the gauge slits, a trapezoid slit profile was used. The diffracted signal was recorded on a 2D-area detector (mar CCD), capable of recording one scan every seven seconds. The detector–sample distance was set to capture the first five Bragg reflections (Debye–Scherrer rings), so enabling a reasonable compromise between recording sufficiently high diffraction orders while still maintaining a good angular resolution. For data analysis, the 2D scans were averaged over 2°-wide polar segments to give 180 radial line scans, which were then fitted by split-Pearson-VII functions to obtain peak parameters such as the 2θ peak position, the integral intensity, and the integral peak width. For more details related to data reduction, see recent work by Lohmiller et al. [11]. Because of the short wave length of the high energy synchrotron beam, the only grains that are diffracting are those that have the diffracting vector g (lattice plane normal) almost normal to the incoming beam direction (deviation Θ = arcsin (λ/2d_(hkl)_): (111): 2.27°, (200): 2.62°, (220): 3.64°, with the Bragg angle Θ and lattice plane spacing d_(hkl)_), Figure 1c. It should also be mentioned that high monochromacy of the synchrotron radiation strongly reduces the fraction of grains in the Bragg condition. Thus, because of the high transmission, synchrotron radiation is an appropriate way to reach high grain statistics to a level reliable for measurements of weak textures, which is very important in the present texture analysis. Note that not all of the crystallographic texture was measured in the experiments because the sample was not rotated. Thus, since the specimen was not rotated, only low scattering angles of the diffraction circles were available, and this allowed for the determination of the peripheral parts of the pole figures only.

### 2.2. Texture Simulations

The texture development due to plastic strain was modeled using the Taylor viscoplastic polycrystal model. This uniform deformation polycrystal approach was justified in several previous studies of the present authors, which proved to be applicable to different nano-polycrystalline materials [1,6]. It was also found in those studies that the major operating slip mechanism was a <112> type partial dislocation slip on {111} planes. The usual {111}<110> slip systems were also considered in the simulations; the relative slip resistance of the two slip system families was kept at 1.5 during deformation ($\tau_{0}^{110}/\tau_{0}^{112}=1.5$). Strain hardening was not considered because nc materials do not show significant strain hardening. The slip rates ${\dot{\gamma }}^{\left(\mathrm{slip}\right)}$ were connected to the resolved shear stresses $\tau^{\left(\mathrm{slip}\right)}$ using the strain rate sensitive slip law of Hutchinson [13]:

(1) $${\dot{\gamma }}^{\left(\mathrm{slip}\right)}={\dot{\gamma }}_{0}{}^{\left(\mathrm{slip}\right)}{\left(\frac{\tau^{\left(\mathrm{slip}\right)}}{\tau_{0}^{\left(\mathrm{slip}\right)}}\right)}^{1/m}.$$

Here ${\dot{\gamma }}_{0}{}^{\left(\mathrm{slip}\right)}$ is a reference rate and m is the strain rate sensitivity index for slip. A relatively high value was used for m (m = 1/6), which can be justified by (i) the rounding effect of the yield surface produced by viscoplastic slip [14] and (ii) the enhanced strain rate sensitivity of nano-polycrystalline materials [15].

Grain boundary sliding (GBS) was taken into account as an additional deformation mechanism of dislocation slip. For a quantitative simulation of the GBS process, the grain boundaries (GBS) were modeled by flat planes in 3D Cartesian space, by constructing a regular dodecahedron with 12 facets for each grain, with different initial positions (see more about the GBS modeling approach in [7]). The deformation generates changes in the orientation of the GBS. To account for this important effect, the directions of the GB normals were updated by the sum of the rigid body rotations of the macroscopic velocity gradient and the one corresponding to the GBS. The constitutive law for GBS was taken similar to that of the slip, with the difference that linear viscous slip was assumed for GBS:

(2) $${\dot{\gamma }}^{\left(\mathrm{GB}\right)}={\dot{\gamma }}_{0}{}^{\left(\mathrm{GB}\right)}\left(\frac{\tau^{\left(\mathrm{GB}\right)}}{\tau_{0}{}^{\left(\mathrm{GB}\right)}}\right).$$

Although the GBS process is considered here linear viscous, considering that the crystallographic slip within the grains is non-linear (Equation (1)), the overall behavior is also non-linear. The resolved shear stress acting on the GBS was calculated from the macroscopic stress tensor ${\underset{=}{\sigma }}^{\left(\mathrm{Mac}\right)}$:

(3) $$\tau^{k(\mathrm{GB})}=\sigma_{\mathrm{ij}}{}^{\left(\mathrm{Mac}\right)}m_{\mathrm{ij}}{}^{k(\mathrm{GB})}.$$

The $m_{\mathrm{ij}}{}^{k(\mathrm{GB})}$ Schmid-type GB orientation tensor was constructed from the GB normal ${\underset{-}{n}}^{k}$ and the GB-slip ${\underset{-}{g}}^{k}$ unit vectors: ${\underset{=}{m}}^{k(\mathrm{GB})}={\underset{-}{g}}^{k}⊗\underset{-}{n^{k}}$. The latter was assumed to be in the direction of the maximum resolved shear stress acting in the GB plane.

From the Schmid orientation tensor ${\underset{=}{m}}^{k(\mathrm{GB})}$ and the sliding rate ${\dot{\gamma }}^{k(\mathrm{GB})}$ of each GBS system, the velocity gradient $L_{\mathrm{ij}}^{\left(\mathrm{GBS}\right)}$ corresponding to the GBS of a grain can be calculated by

(4) $$L_{\mathrm{ij}}^{\left(\mathrm{GBS}\right)}=\sum_{k=1}^{12}m_{\mathrm{ij}}{}^{k(\mathrm{GB})}{\dot{\gamma }}_{i}^{k(\mathrm{GBS})}.$$

The grain interior is subjected to the difference between the macroscopically imposed and GBS-produced gradient tensor:

(5) $$L_{\mathrm{ij}}^{\left(\mathrm{grain}\right)}=L_{\mathrm{ij}}^{\left(\mathrm{Mac}.\right)}-L_{\mathrm{ij}}^{\left(\mathrm{GBS}\right)}.$$

This velocity gradient $L_{\mathrm{ij}}^{\left(\mathrm{grain}\right)}$ was used to solve the crystal plasticity problem in full constraints Taylor deformation mode to obtain the slips in each slip system and then the lattice orientation change, from which the texture evolution can be calculated. Fifty thousand grains were considered in the simulations with initially perfectly random orientation distribution. The maximum applied compressive strain was 0.27, ideally yielding a maximum shear strain of 0.54. The texture simulations were done in strain steps of 0.01.

Note that the experiments were planned for simple shear tests; however, the experimental setup led to deviation from the ideal simple shear conditions. Namely, during compression, the length of the gauge section was reduced leading to an axial compression strain component. Therefore, additional to the ideal simple shear velocity gradient ${\underset{=}{L}}_{}^{\left(\mathrm{shear}\right)}$, a plane strain type velocity gradient had to be considered:

(6) $${\underset{=}{L}}^{\left(\mathrm{Mac}.\right)}={\underset{=}{L}}^{\left(\mathrm{shear}\right)}+{\underset{=}{L}}^{\left(\mathrm{comp}.\right)}.$$

As the simulations were done in the experimentally defined reference system, the following macroscopic velocity gradient tensor was used:

(7) $$L_{\mathrm{ij}}^{\left(\mathrm{Mac}.\right)}=\left|\dot{\gamma }\right|\left(\begin{array}{ccc} 0.5 & -0.5 & 0\\ 0.5 & -0.5 & 0\\ 0 & 0 & 0 \end{array}\right)+\left|\dot{\varepsilon }\right|\left(\begin{array}{ccc} 0 & 0 & 0\\ 0 & -1 & 0\\ 0 & 0 & 1 \end{array}\right).$$

Here the first term on the right side is the simple shear part—operating at 45° with respect to the x–y–z reference system in the x–y plane—and the second part is the plane strain compression part, see Figure 1 for the definition of the reference system. The effect of the compressive strain is very significant on the texture evolution, so its relative proportion to the shear was varied by the parameter $C=\left|\dot{\varepsilon }\right|/\left|\dot{\gamma }\right|$. The following values were employed: C = 0, 0.15, 0.3, and 0.45. Finite element simulations from [16] suggested that the most probable value is C = 0.3. As shown below, our simulations indeed led to the best agreement with the experiments when C was 0.3.

## 3. Results and Discussion

**Figure 2:**
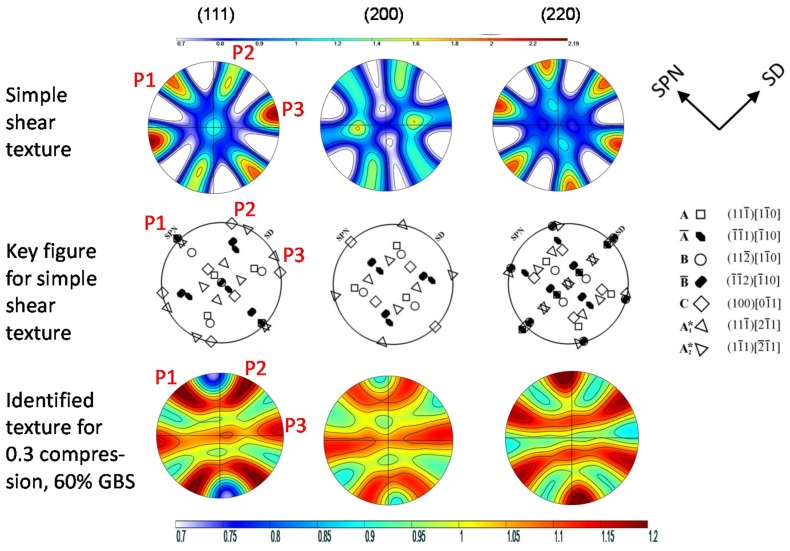
Pole figures simulated for simple shear and 60% grain boundary sliding (GBS) with C = 0.3. Maxima at the periphery evaluated are marked with P1, P2, and P3. The main texture components of simple shear deformed fcc metals (denoted by {hkl} parallel to the shear plane and <uvw> parallel to the shear direction) are given in the key figure.

**Figure 3:**
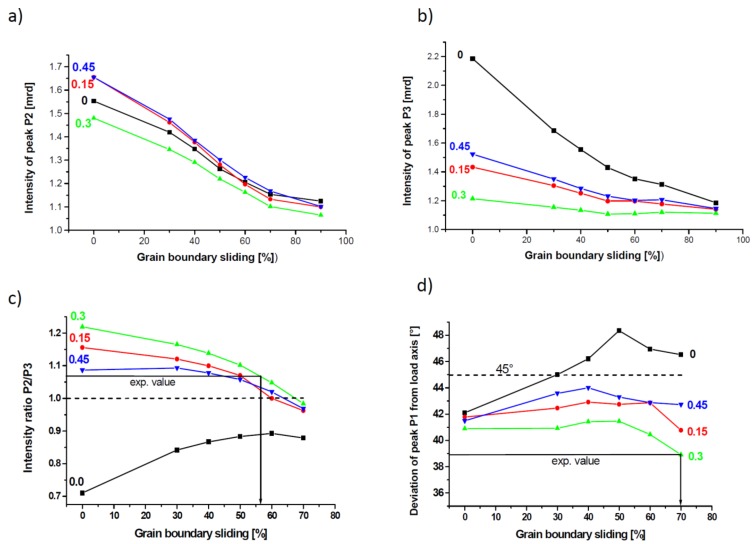
Dependence of the peak intensities P2 (**a**) and P3 (**b**), intensity ratio P2/P3 (**c**), and angular deviation of Peak P1 from the load axis (**d**) on the contribution of GBS to strain with the fraction of compressive strain as parameter. In (c,d), the amount of GBS is estimated from the experimentally measured parameters.

Typical pole figures for two cases, simple shear and a combination of simple shear and compression (C = 0.3) plus an additional 60% of GBS, are shown in Figure 2 together with the key figure of the typical components developing during the simple shear of fcc metals (e.g., [1]). The simple shear texture at low strain is composed of all main components, in contrast to severe plastic deformation by high pressure torsion (HPT), where a dominant B/$\bar{B}$ component was found [1]. The contribution of the compressive strain leads to a tilted <110> fiber texture, while GBS leads to texture weakening. For a quantitative analysis, the intensities and positions of the maxima marked at the edge (outer great circle) of the (111) pole figure are plotted as a function of GBS and a fraction of compressive strain in Figure 3. The intensities of Peak P2 (Figure 3a) and Peak P3 (Figure 3b) evolve with increasing GBS approaching 1.0 mrd, i.e., the texture becomes randomized. At constant amount of GBS, the intensity is lowest for C = 0.3. As the absolute intensity depends on the angular Gaussian spread used (15° was imposed), the intensity ratio of Peaks P2 and P3 has been chosen instead (Figure 3c). This ratio is greater or smaller than 1.0 and changes with the amount of GBS and compressive strain. As Peaks P2 and P3 are related to the shear texture components C + A_1_* and C + A_2_* (see key figure of Figure 2), respectively, it is demonstrated that GBS affects the texture components differently. 

Another interesting result is that GBS changes the position of Peak P1. This peak is related to components of the so-called A-fiber, namely A_1_*, A_2_*, A, and $\bar{A}$, which are characterized by the alignment of the (111) plane parallel to the shear plane, i.e., its normal is positioned anticlockwise at 45° with respect to the loading direction (Figure 2). However, with an increasing amount of GBS for C = 0, an overshooting takes place (Figure 3d). Indeed, for the simple shear, the texture components tilt opposite to the shear direction with respect to their ideal positions if GBS is high [7].

**Figure 4:**
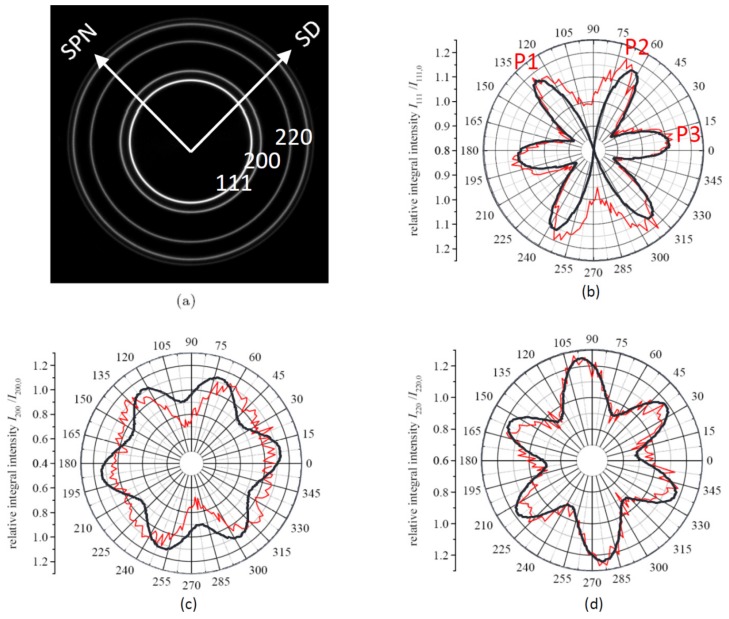
Experimentally measured intensity profile along the Debye–Scherrer rings (**a**) for the (111) (**b**), (200) (**c**), and (220) reflections (**d**) at a compressive strain of 0.27 applied to the gauge section in a load direction normalized with the intensity of the undeformed sample (taken from [5]). Overlaid are the simulated intensity profiles.

The results of the simulations and those of the experiment are shown in Figure 4. This figure shows rose diagrams (Figure 4b–d) of the intensity along the Debye–Scherrer rings (Figure 4a) for a single fixed sample orientation as a function of the compressive strain applied to the gauge section in the loading direction, i.e., the intensity profile along the outer circle of the pole figures (with small deviation of Θ, see above and Figure 1), so a “partial texture” can be seen. Overlaid is the simulated best fitting intensity profile at maximum strain applied for 60% GBS and C = 0.3. There is a quite good fit for the (111) and (220) reflections, but less agreement for (200), where only the experimental minima are captured. However, this disagreement should not be overvalued, because the (200) reflection has the lowest experimental intensity due to the lowest plane multiplicity. A GBS amount of 55% and 70% can also be estimated based on data shown in Figure 3c and Figure 3d, respectively. These values depend on the fraction of plane strain compression and are due to the semi-controlled boundary conditions of the simple shear applied to the sample. Good agreement was found for C = 0.3, which is also the value estimated from finite element simulations of the deformation process [16]. Previously, a smaller value (30%) was found in a texture simulation [1] with a larger grain size (>20 nm) due to stress-induced grain growth during large-strain HPT. This clearly shows the effect of grain size on the amount of GBS.

## 4. Conclusions

Based on texture simulations and a comparison with an experiment, the following conclusions can be drawn:

(i) GBS decreases the texture strength but keeps the signatures of the texture type.
(ii) GBS affects the texture components differently with respect to intensity and angular position.
(iii) The amount of GBS can be estimated from the texture evolution as a function of GBS.
(iv) In the investigated Pd–10 atom % Au alloy with a grain size of about 15 nm, GBS is the predominant deformation mode at room temperature, contributing to strain by about 60%.
